# Tracing Makerere University's 100 years' contribution to lung science/medicine

**DOI:** 10.4314/ahs.v22i2.9S

**Published:** 2022-08

**Authors:** Bruce J Kirenga, Jasper Nidoi, Winters Muttamba, Simon Walusimbi, Rejani Lalitha, Harriet Mayanja-Kizza, Samuel Yoo, Joseph Imoko, Joseph Kawuma, William Worodria

**Affiliations:** 1 Department of Medicine, School of Medicine, Makerere University College of Health Sciences, Kampala, Uganda; 2 Makerere University Lung Institute, Kampala, Uganda; 3 Department of Internal Medicine, Institute of Health Jimma University, Jimma, Ethiopia; 4 Former TB Advisor, WHO Country Office, Kampala, Uganda; 5 Former Medical Advisor, German Leprosy and TB Relief Association Uganda Office, Kampala, Uganda

## Abstract

Dear Editor, African Health Sciences Journal,

This year, 2022, Makerere University will be celebrating 100 years of existence. As current lung science/medicine experts, we felt it as important to trace the University's contribution to lung science and medicine. In this letter, we trace and describe some of the early work done by Makerere University/Mulago Hospital affiliated scientists, identify prominent players in lung science over the 100 years, and present the university's scholarly contribution to this field, as available in online databases. We include both Makerere University and Mulago Hospital affiliated scientists, because for many years, staff of these two institutions have worked together in teaching, research and patient care.

## History

Makerere University's contribution to lung science dates back to the works of the co-founder of Makerere University medical school and Mulago Hospital, Sir Albert Ruskin Cook. In a paper published by T. M. Daniel in the international Journal of Tuberculosis and Lung Diseases, Daniel found that Albert Cook was involved in diagnosis and treatment of TB at Mengo Hospital in the 1800s^1^. It was noted that Cook had two microscopes that he had carried with him to Uganda. As can be seen in this sample case record, Figure 1A, Cook made a diagnosis of phthisis which is the medieval name for TB. Later on, Cook went on to discover another disease closely related to Mycobacterium TB called Buruli ulcer whose causative organism (Mycobacterium Ulcerans) was isolated in 1948 by Peter MacCallum in the Bairnsdale region of Victoria, Australia. After this nothing much can be found until the 1980s where work on TB started emerging due to the outbreak of the HIV pandemic. In 1993 Dr. Martin Okot-Nwang published a paper describing increasing cases of TB in Mulago Hospital in the period 1985–1989 2. This paper was one of the earlier papers to show the link between increasing TB prevalence and HIV globally.

**Figure:**
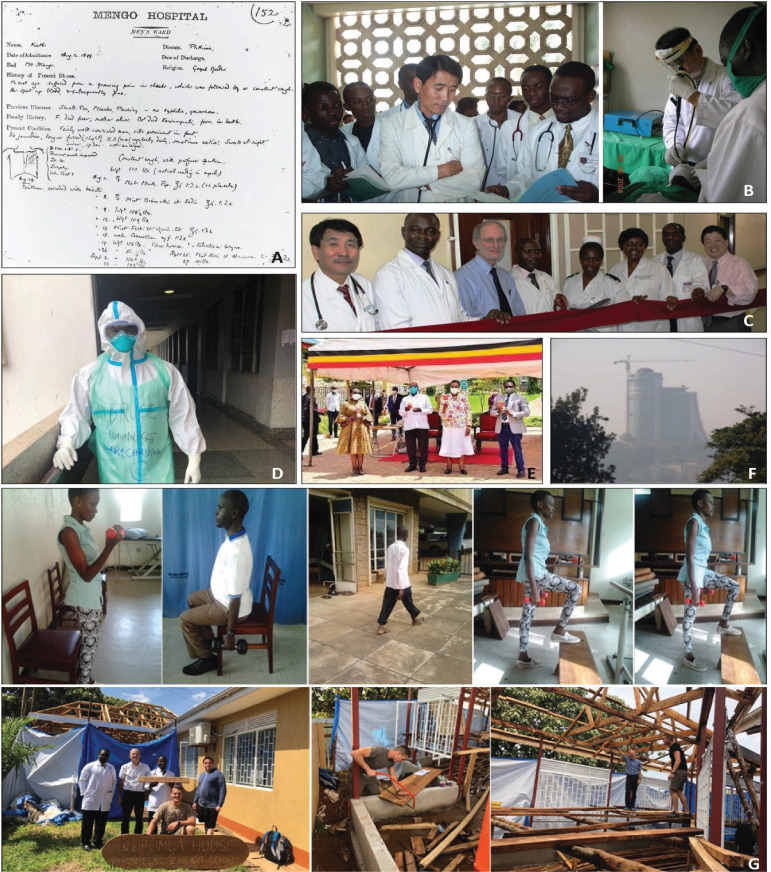
**A:** A representative admission notes by Albert Cook describing a male patient with pulmonary tuberculosis. A history of cough, hemoptysis, and night sweats is recorded. As is typical of Cook's notes, the physical findings over the left upper lobe. **B:** Dr. Yoo on teaching ward round ward 4C of Muiago, also seen is Dr. Bruce Kirenga, then an intern in medicine -shared by Dr. Yoo. **C:** Dedication of the Bronchoscopy Suite at Mulago Hospital. **D:** Dr. Bruce Kirenga setting off for COVID word-Christmas 2020. **E:** Launch of UBV trial by His Excellence Yoweri Kaguto Museveni (President of the Republic of Uganda). **F:** Photo taken during the conduct of air pollution in Kampala. **E:** Photo montage of PR in pictures

## Faces of lung science/medicine at Makerere University

Earlier contributors to lung science at Makerere University are hard to come by. In the 1970s to 1980s, Dr. Dee who worked on the TB ward, is believed to have trained Dr. Peter Eriki in the practice of pulmonary medicine. Dr. Eriki was subsequently instrumental in establishing the Uganda National Tuberculosis Programme which later merged with the National Leprosy Programme birthing the current National Tuberculosis and Leprosy Programme (NTLP). Indeed, he was the first programme manager of the NTLP. Dr. Eriki later joined the World Health Organization (WHO) as part of his career journey. Later Dr. Okot-Nwang became the prominent face of lung science/medicine after Dr. Eriki. He taught most of current lung scientists/physicians in the country. Dr. Okot-Nwang worked alongside Dr. Alphonse Okwera (deceased), who mainly pushed forward the TB reserch agenda. Lung science/medicine also received contribution from Dr. Samuel Yoo, a Korean missionary doctor, who tremendously contributed to teaching and pulmonary care, Figure 1B. He is especially credited for introducing bronchoscopy (1992), chest ultrasound and pulmonary function testing (2005) in the Department of medicine.

Dr. Yoo and Martin Okot-Nwang taught Dr William Worodria mastery in pulmonary medicine, especially in interventional pulmonology/bronchoscopy, which he still runs to date. Dr Worodria has taught most of the current pulmonologists including Dr Simon Luzige (current Director of Nakasero Hospital), Dr. Bruce Kirenga, Dr. Lydia Nakiyingi, Dr. Rejani Lalitha, Dr. Ivan Kimuli, Dr. Patricia Alupo, Dr. Winceslaus Katagira, Dr. Susan Adakun and Dr. Joseph Baluku. In 2006, Prof Harriet Mayanja-Kizza as Head of Department of Medicine introduced sub-specialized medicine units, making the department the first to get specialized. One of the units created was the Pulmonology Unit, with the pioneer Head as Dr. Sam Yoo.

In public health and research Dr. Francis Adatu took charge of TB control at the Ministry of Health. Although not a lung scientist per se, Prof. Roy Mugerwa made huge contributions to TB research. Working with the Case Western University Research Collaboration together with Prof. Joloba (current Dean of the School of Biomedical Sciences), Prof. Harriet Mayanja-Kizza (former Dean of the school of Medicine) among others, they set up probably, the first formal TB research unit on in the country. The history of this unit, is posted on the CWRU website: (https://case.edu/medicine/tbru/about-us/history)

Another notable collaboration that immensely contributed to pulmonary care and research is the Makerere University – University of California San Francisco (UCSF) Research Collaboration which is credited with contributing to research into HIV associated lung complications^3^ and the building of a state-of-the-art bronchoscopy service in the country; see Figure 1C showing the launch of the bronchoscopy suite.

## The birth of the Makerere University Lung Institute

In 2015, the Makerere University Lung Institute (MLI) was founded with the main aim of championing lung science in the university. Since its founding, the institute has had significant contribution to lung science by expanding the number of lung scientists, publications, and building diagnostic and care capacity. The five-year impact of the institute is presented as a supplementary appendix. The institute has also been instrumental in the response to COVID-19 in terms of frontline patient management and research^4^–^7^; see Figure 1D and 1E.

For many years Uganda did not have data on the burden of asthma and COPD. Since its founding, together with collaborators, MLI has conducted landmark studies on asthma and COPD that have provided data to the Ministry of Health for programming.

Another landmark study was the pioneer work on air pollution in the country. Prior to this study, there was completely no data on air pollution in the country. Scientists at Makerere University in collaboration with Yale University designed and conducted the first air pollution study in Uganda which showed that the air quality was very poor^8^, see Figure 1F This study provided evidence for follow on studies and formation of policies by the city authorities.

MLI started pioneer work on pulmonary rehabilitation (PR) for patients with permanent lung damage from TB. Previously, this treatment modality was mainly used for COPD and other chronic lung diseases and had never been tested in post TB patients. However, PR care was limited by the absence of appropriate equipment in Uganda which is available in developed settings. Therefore, PR practitioners improvised by using benches and recycled mineral water bottles filled with tap water as weights in many cases, see Figure 1G.

The results from the pilot projects were amazing; bed ridden patients went back on their feet^9^. These results supported acquisition follow on large grants including the FRESHAIR project (UK, Uganda, Vietnam, Kyrgyzstan, Greece, https://www.ipcrg.org/freshair) and Global Recharge (UK, Uganda, Siri Lanka and India, https://www.leicesterbrc.nihr.ac.uk/themes/respiratory/research/global-recharge/) and expansion of infrastructure through crowd funding. One of the crowd donors flew into the country to physically participate in the construction as can be seen in Figure 1G.

Very little has been done on lung cancer in Uganda with the exception of work done by Prof Wabinga as part of the Kampala cancer registry. More recently the Lung Institute in collaboration with CWRU scientists has won a large grant to study lung cancer in Uganda and Tanzania.

MLI runs several programs through its Department of Education and Training such as medical rotations, Day of Lung Science held once a month, capacity building programmes and specialised training sessions (bronchoscopy, smoking cessation, COVID-19, sleep trainings, spirometry). It has trained over 2489 scientists and clinicians including 507 health workers from 56 districts who were trained on COVID-19 case management protocols, infection prevention and control and home-based care. A Pulmonary and Critical Care Medicine Program was developed in collaboration with the department of medicine and is undergoing review for approval. It aims to train superspecialists in pulmonology under two tracks: a 2-year fellowship and a 4-year medical degree.

## Scholarly contribution

**Figure 2:**
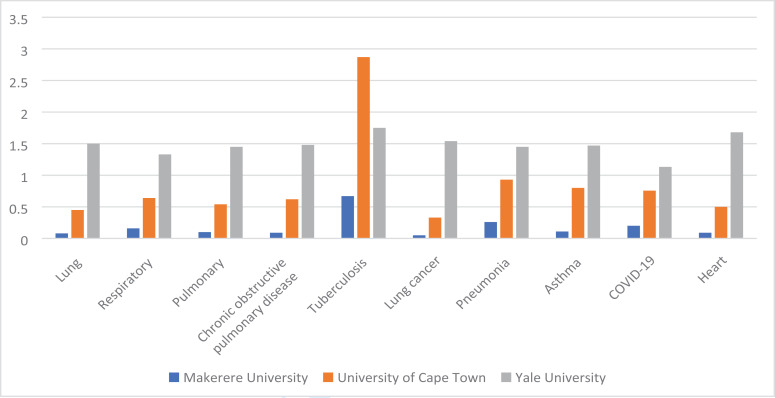
Hits returned in PubMed central on December 4 16–17.13 Hours on searching of the terms and respective universities, % of total hits

To trace the contribution of Makerere University to research in lung science in recent times, we performed a search in the PubMed online database on 4th December 2021 between 16:00–17:13 hours for terms related to lung science (lungs, respiratory, pulmonary) or major lung diseases and Makerere University (Mak) without restriction on year of publication. We also searched for the same words for University of Cape Town (UCT) and Yale University for comparison. In addition, we searched for “heart” to compare across organs. The full list of terms and corresponding yields per university of this search are summarized in Table 1 and Figure 2. The search showed that scholars affiliated with Makerere University had made substantial contributions to research in lung science. However, the contribution of Makerere University paled when compared to UCT and Yale University. Furthermore, as can be seen in the table and figure, research in the heart field outperformed lung science indicating that more efforts should be made to attract scientists to lung science.

**Table 1:** Hits returned in PubMed central by search terms and respective university

TERM	ALL HITS	MAKERERE UNIVERSITY		UNIVERSITY OF CAPE TOWN		YALE UNIVERSITY	
		No.	%	No.	%	No.	%
**LUNG**	1202238	950	0.08	5461	0.45	18086	1.5
**RESPIRATORY**	997935	1593	0.16	6368	0.64	13303	1.33
**PULMONARY**	1553347	1591	0.1	8427	0.54	22453	1.45
**COPD**	168709	157	0.09	1039	0.62	2492	1.48
**TUBERCULOSIS**	315652	2124	0.67	9068	2.87	5537	1.75
**LUNG CANCER**	753026	336	0.05	2478	0.33	11608	1.54
**PNEUMONIA**	409848	1067	0.26	3823	0.93	5943	1.45
**ASTHMA**	266140	297	0.11	2125	0.8	3908	1.47
**COVID-19**	270290	543	0.20	2045	0.76	3062	1.13
**HEART**	1476282	1296	0.09	7339	0.5	24866	1.68

## Future perspectives- the next 100 years

In the coming decades, respiratory conditions due to infectious diseases, malignancies, allergic diseases, autoimmune disorders, and occupational diseases are expected to cause increased morbidity and mortality worldwide. Makerere University will therefore need to position itself at the forefront of the research efforts in the prevention and treatment of this wide range of respiratory diseases. To facilitate this process, the University will need to expand research, teaching and clinical care infrastructure. Dedicated efforts are needed to attract the best brains in lung science.

## Figures and Tables

**Supplementary File 1:**
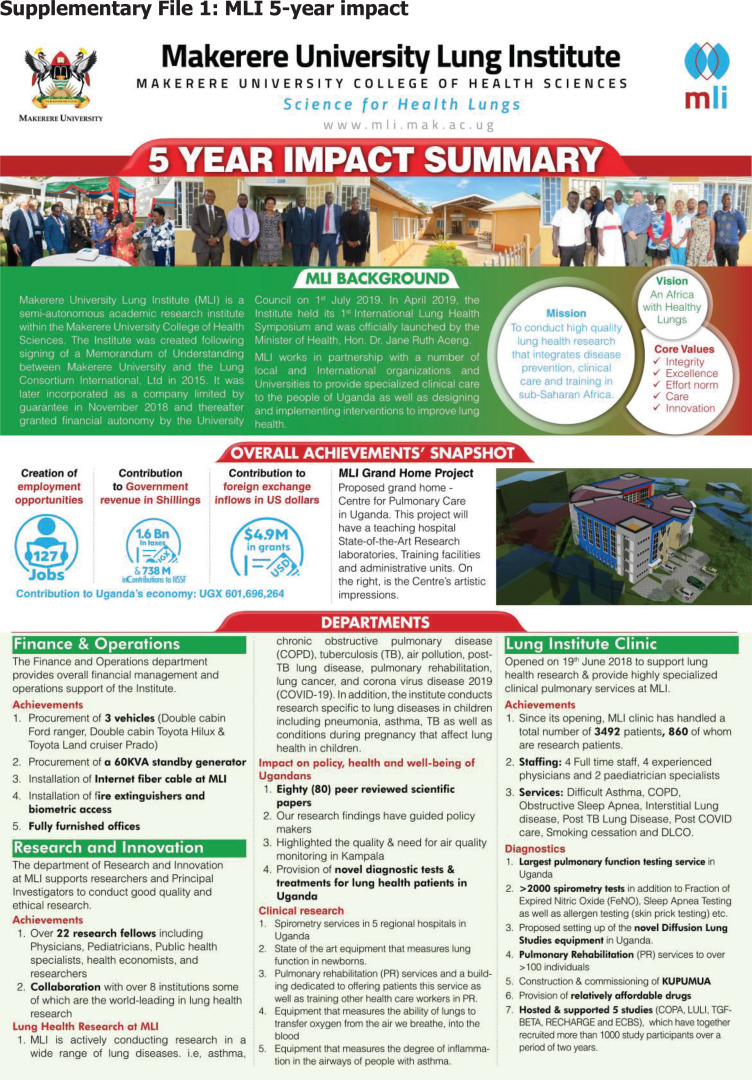
MLI 5-year impact
